# Structure of a Novel Winged-Helix Like Domain from Human NFRKB Protein

**DOI:** 10.1371/journal.pone.0043761

**Published:** 2012-09-11

**Authors:** Abhinav Kumar, Sabine Möcklinghoff, Fumiaki Yumoto, Lukasz Jaroszewski, Carol L. Farr, Anna Grzechnik, Phuong Nguyen, Christian X. Weichenberger, Hsiu-Ju Chiu, Heath E. Klock, Marc-André Elsliger, Ashley M. Deacon, Adam Godzik, Scott A. Lesley, Bruce R. Conklin, Robert J. Fletterick, Ian A. Wilson

**Affiliations:** 1 Joint Center for Structural Genomics, La Jolla, California, United States of America; 2 Stanford Synchrotron Radiation Lightsource, SLAC National Accelerator Laboratory, Menlo Park, California, United States of America; 3 Department of Biochemistry and Biophysics, University of California San Francisco, San Francisco, California, United States of America; 4 Department of Molecular Biology, The Scripps Research Institute, La Jolla, California, United States of America; 5 Protein Sciences Department, Genomics Institute of the Novartis Research Foundation, San Diego, California, United States of America; 6 Program on Bioinformatics and Systems Biology, Sanford-Burnham Medical Research Institute, La Jolla, California, United States of America; 7 Center for Research in Biological Systems, University of California San Diego, La Jolla, California, United States of America; 8 Gladstone Institute of Cardiovascular Disease, San Francisco, California, United States of America; 9 Departments of Medicine and Cellular and Molecular Pharmacology, University of California San Francisco, San Francisco, California, United States of America; Institute of Enzymology of the Hungarian Academy of Science, Hungary

## Abstract

The human nuclear factor related to kappa-B-binding protein (NFRKB) is a 1299-residue protein that is a component of the metazoan INO80 complex involved in chromatin remodeling, transcription regulation, DNA replication and DNA repair. Although full length NFRKB is predicted to be around 65% disordered, comparative sequence analysis identified several potentially structured sections in the N-terminal region of the protein. These regions were targeted for crystallographic studies, and the structure of one of these regions spanning residues 370–495 was determined using the JCSG high-throughput structure determination pipeline. The structure reveals a novel, mostly helical domain reminiscent of the winged-helix fold typically involved in DNA binding. However, further analysis shows that this domain does not bind DNA, suggesting it may belong to a small group of winged-helix domains involved in protein-protein interactions.

## Introduction

The INO80 complex is a universally conserved multi-subunit protein complex anchored around the Snf2 family ATPase (INO80 protein), and is involved in several DNA-related functions including chromatin remodeling, transcription regulation, replication, and DNA repair [1]–[7]. The mammalian INO80 complex is composed of three protein modules [2], one of which consists of proteins specific to metazoa (animals), and not involved in ATP-dependent nucleosome remodeling. Nuclear factor related to kappa-B-binding protein (NFRKB) is a part of this module and modulates the deubiquitinase activity of UCHL5 in the INO80 complex [7]. Recent genome-wide RNAi screening revealed that NFRKB has an important function in the acquisition of pluripotency of human cells [8]. NFRKB enhances induced pluripotent stem cell generation and knockdown of the NFRKB gene affects the reprogramming process leading to a reduced number of human induced pluripotent stem (iPS) cell colonies.

The human NFKRB protein (Uniprot ID Q6P4R8), also known as subunit G of the INO80 complex, consists of 1299 amino acids. This subunit is responsible for DNA binding with a consensus sequence of 5′-GGGGAATCTCC-3′ [7]. The structure of the full-length NFRKB has yet to be determined, most likely due to the challenges of crystallizing such a large protein with numerous domains and predicted disordered regions. NFRKB is predicted to be 65% disordered, with the longest disordered segment (650aa) spanning the entire C-terminal half of the protein. However, at least three structured domains are predicted in the N-terminal half of NFRKB, and could be amenable to X-ray structural studies [9]. Based on PsiPred and Disopred sequence analyses, we made 16 constructs that covered various possible boundaries of these three putative domains, and one construct, residues 370–495, produced diffraction-quality crystals. The structure of this domain was determined to 2.18 Å resolution and reveals a novel helical domain (NFRKB_WHL) bearing remarkable similarity to a winged-helix domain, usually associated with DNA binding. Based on our structural findings, this domain was subsequently used to seed a new NFRKB winged-helix-like PFAM [10] family (PF14465).

## Results and Discussion

### Overall Structure

The crystal structure of the human NFRKB_WHL domain (residues 370–495) consists of two protomers (residues 372–483 in chain A and 370–483 in chain B), one sodium ion and 25 water molecules in the crystallographic asymmetric unit. The last 12 residues of the construct (residues 484–495) were disordered and were not modeled. In addition, residues 370–371, 463–465 in chain A and 440–443, 462–463 in chain B were not modeled due to poor electron density. The Matthews' coefficient (V_M_) [11] and the estimated solvent content are 1.84 Å^3^/Da and 33.1% respectively. The Ramachandran plot produced by *MolProbity* [12] shows that 97.5% of the residues are in favored regions with no outliers.

**Figure 1:**
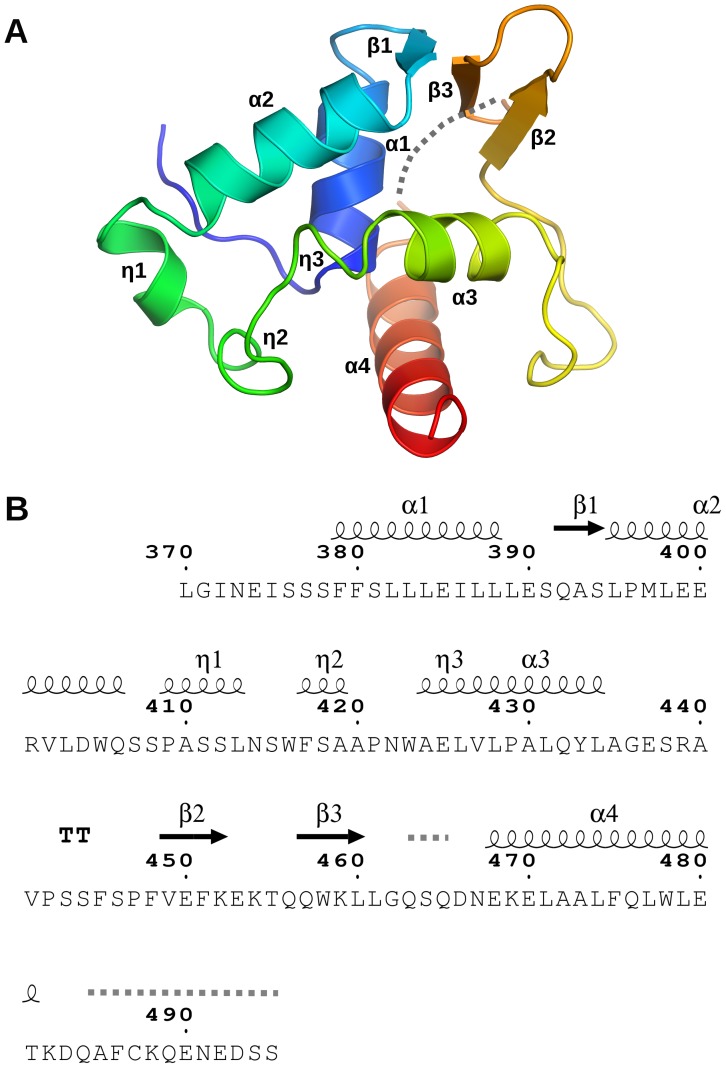
Crystal structure of the winged-helix domain of human NFRKB. (**A**) Ribbon diagram of the human NFRKB_WHL domain (residues 370–495) structure is color-coded from N-terminus (blue) to C-terminus (red). Helices α1–α4, β-strands β1–β3 and 3_10_ helixes η1–η3 are indicated. The dashed line between β3 and α4 corresponds to three disordered residues that were omitted from the model. (**B**) Protein sequence of the NFRKB_WHL domain annotated with the corresponding secondary structure elements. The dashed lines indicate residues that were in the construct, but are not in the refined model due to lack of interpretable electron density. Figure 1B was prepared with ESPript [40].

The NFRKB_WHL structure adopts a fold similar to the winged-helix DNA binding fold, comprising of four α-helices, three 3_10_-helices and a short β-sheet composed of three β-strands (Figure 1).

### Structural similarities to other DNA binding proteins

A search for structurally similar proteins using the DALI server [13] returned about 300 hits with Z-scores above 5.0, with a maximum score of only 7.5. Most of the matches were to DNA binding domains within large, multidomain, proteins, with a few low scoring matches to proteins involved in protein-protein interactions. The closest match was to the DNA binding domain of the virulence gene activator AphA protein from *Vibrio cholerae* (PDB code 1yg2 [14]) with a Z-score of 7.5 and RMSD of 3.0 Å over 67 residues. This structure, however, was determined without DNA and, therefore, does not provide any insights on the potential role of NFRKB _WHL in DNA binding.

### Can NFRKB_WHL bind DNA?

**Figure 2:**
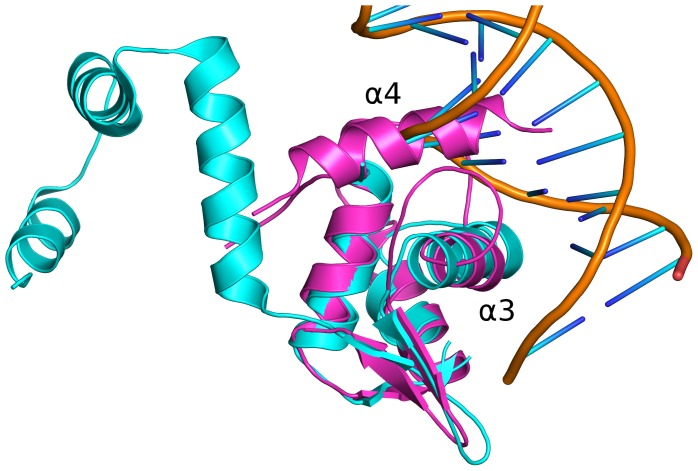
Superposition of NFRKB_WHL domain structure with the MecI repressor structure from *Staphylococcus aureus* (PDB code 2d45). Helix α3 of the NFRKB_WHL domain (magenta) is located in the major groove of the DNA upon superposition onto the MecI repressor (cyan, protein and orange, DNA backbone). Helix α4 of NFRKB_WHL does not map to any corresponding helix in MecI repressor and clashes with the DNA.

**Figure 3:**
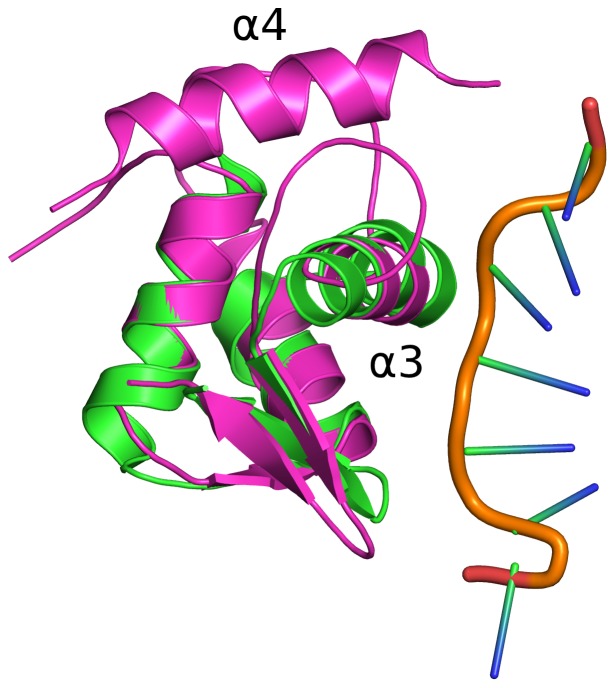
Superposition of NFRKB_WHL domain onto the DNA-binding domain Zα of DLM-1 (PDB code: 1j75). The DNA binding domain Zα of DLM-1 (green) binds a left-handed Z-DNA (orange backbone) and has limited interactions with the DNA. The human NFRKB_WHL domain is shown in magenta with α3 and α4 helices labeled.

In order to investigate whether NFRKB_WHL might interact with DNA, 45 of the top DALI hits that had bound DNA in their structures were superimposed onto the NFRKB_WHL structure to identify regions potentially involved in DNA binding (Figure 2). Further analysis of the superimposed structures revealed two types of DNA binding modes. The first group includes structures where the main recognition helix (α3 in NFRKB_WHL) binds in the major groove of the bound DNA (PDB codes 2d45, 1sax, 1u8r, 1xsd, 2xro, 3co7) (Figure 2). The second group includes structures with Z-DNA bound to proteins (PDB codes 1j75, 2gxb, 2heo, 3eyi) where there is limited interaction between protein and DNA (Figure 3).

**Figure 4:**
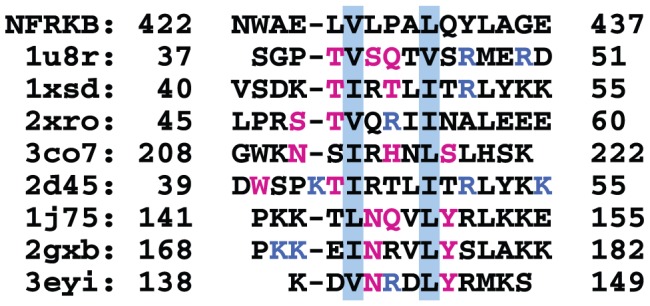
Structure-based sequence alignment of helix α3. Helix α3 of NFRKB_WHL was aligned with the corresponding helices from some of the structurally similar proteins based on DALI alignment. Only two hydrophobic residues of NFKRB (427 V and 431 L; blue-grey background) exhibit some degree of conservation. Residues interacting with DNA in other structures are colored (polar and aromatic residues in pink and basic residues in blue).

**Figure 5:**
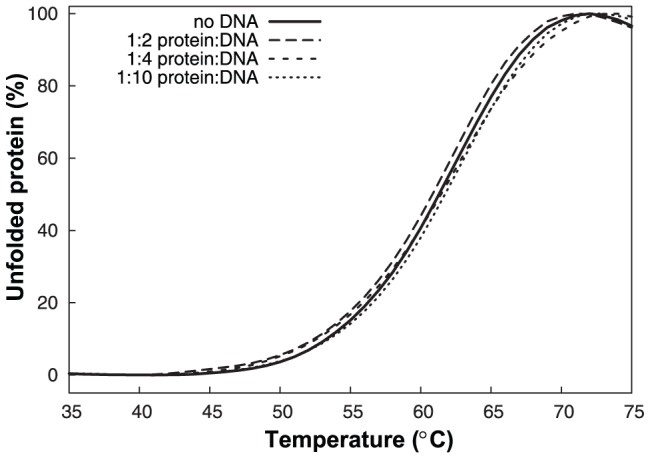
Lack of DNA binding to the NFRKB winged helix domain. Comparison of the DSF melting curves of NFRKB winged helix domain in the absence (solid line) and presence of different concentrations of DNA (dashed and dotted lines). The fluorescence of the dye was monitored as a function of temperature. The melting temperature of the protein correlates to amount of binding of the fluorescent dye to the protein as it unfolds. The curves have been normalized setting the maximal/minimal fluorescence response as 0% to 100% protein unfolding.

In both cases, however, the residues interacting with DNA are not conserved in NFRKB_WHL. A structure-based sequence alignment of helix α3 shows a clear lack of conserved residues (Figure 4), with the exception of two hydrophobic residues that point toward the hydrophobic core of the protein and are likely involved in stabilizing the interaction and orientation of this helix on the protein. The sequence of this helix lacks any basic residues, thereby making it unlikely to interact with DNA. Therefore, despite the structural similarity to winged-helix DNA binding domains, NFRKB_WHL is unlikely to bind DNA. The calculated isoelectric point of 4.3 of this domain is also not favorable for DNA binding. In addition, results of Differential Scanning Fluorimetry (DSF) experiments to test whether there is any change in stability of NFRKB_WHL in the presence of consensus DNA (5′-GGGGAATCTCC-3′) further support the observation that NFRKB_WHL may not bind DNA. The protein's melting temperature remains unchanged upon mixing with DNA for different ratios of protein:DNA (Figure 5, Table 1), indicating that the DNA tested does not stabilize NFRKB_WHL.

**Table 1:** Effect of DNA on the thermal unfolding of the NFRKB winged helix domain.

Protein/DNA	Apparent TM (°C)±∼0.1
protein (no DNA)	61.3
protein:DNA ratio 1∶2	61.3
protein:DNA ratio 1∶4	61.3
protein:DNA ratio 1∶10	61.3

The calculated melting temperatures (TM) are mean values of three independent experiments.

### Protein-protein interaction

**Figure 6:**
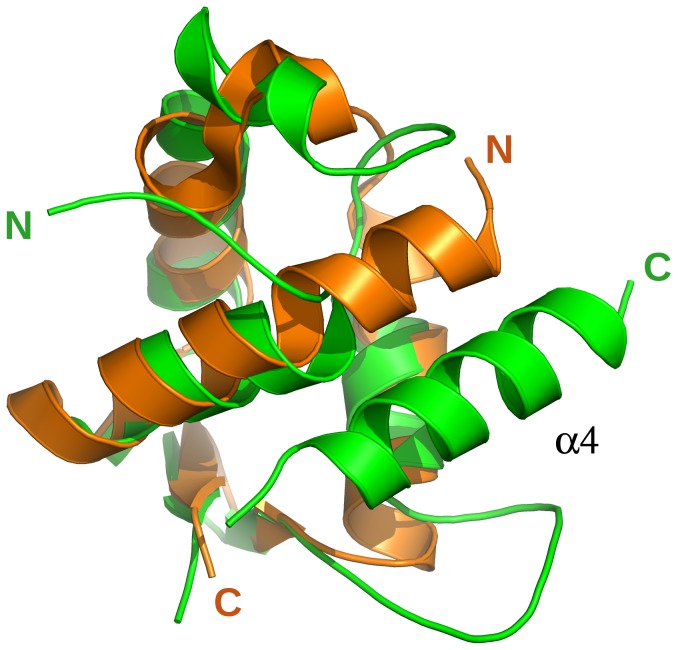
Comparison of NFRKB_WHL domain with the Cul1 domain of the yeast anaphase-promoting complex. Superposition of NFRKB_WHL (green) onto the cullin-homology domain (Cul1) of the anaphase-promoting complex (pdb code: 1ldd, orange) in shown in ribbon representation, with their N- and C-termini labeled. The last helix, α4, in the NFRKB_WHL does not have a counterpart in 1ldd.

Interestingly, one of the higher scoring structural matches identified in the DALI search is a winged-helix domain from the yeast (PDB code 1ldd) and human (PDB code 1ldj) anaphase-promoting complex. NFRKB_WHL has a significant structural similarity (Dali Z-score of 6.7) to one of the winged-helix motifs within the C-terminal domain (CTD) of the cullin protein portion of the of the Cul1–Rbx1–Skp1–F boxSkp2 SCF ubiquitin ligase complex. This domain follows three repeats of the cullin repeat and is involved in binding of the RING finger protein Rbx1 [15]. NFRKB_WHL and the winged-helix subdomain of CTD superpose with a RMSD of 2.15 Å over 63 residues (Figure 6) and share a sequence identity of 10%. Thus, it is possible that NFRKB_WHL may be involved in protein-protein interactions rather than DNA binding.

### New PFAM domain

Initial sequence analysis of this domain, including a sequence search of the PFAM database [10], did not identify any similarity to other winged-helix DNA binding domains. In fact, none of the fold prediction or distant homology recognition tools yielded any statistically significant matches to any characterized protein family. Thus, the NFRKB_WHL structure formed the basis for a new Pfam domain, PF14465. This new domain was thereafter identified in all animal genomes, as well as several single cell eukaryotes. It is always found in proteins bearing overall homology to NFRKB.

## Conclusion

We have determined the structure of a predicted ordered domain from the human NFRKB protein at 2.18 Å resolution. The identification of this domain was based on PsiPred and Disopred sequence analysis that indicated the existence of a structurally ordered region bordered by disordered/low complexity regions. The crystal structure of this domain unexpectedly revealed similarity to winged-helix DNA binding domains in structures, such as MecI repressor (PDB code 2d45; [16]) and Zα domain of DLM-1 (PDB code 1j75; [17]). However, the lack of sequence similarity between this domain and other winged-helix DNA binding domains, the absence of any observable protein-DNA interaction in a DSF experiment, and the lack of positively charged residues in the putative DNA binding helix α3, indicate that this domain likely does not bind DNA. However, similarity to the C-terminal domain in cullins, which is involved in binding of other members of the Skp, Cullin, F-box containing (SCF) complex, suggest a possible role in protein-protein interactions. The NFRKB_WHL domain is the founding member of a new Pfam winged-helix-like family, (NFRKB_winged; PF14465; http://pfam.sanger.ac.uk/family/PF14465), which currently contains 39 sequences from 33 species.

## Materials and Methods

### Domain prediction and construct design

The protein encoded by the human NFRKB gene (Uniprot id: Q6P4R8) did not have any domain annotation in the PfamA database (release 25.0) [10], and fold recognition algorithms (HHPred [18] and FFAS [19]) did not yield any significant domain predictions. However, secondary structure and structural disorder predictions calculated with PsiPred [20] and Disopred [21], respectively, suggested that the N-terminal half of this protein likely contained up to three domains with well-defined, three-dimensional structure. Since these predictions in general do not provide reliable domain boundaries, 16 constructs corresponding to predicted ordered regions were selected (1–208, 1–225, 1–275, 1–307, 1–486, 1–495, 1–651, 1–710, 335–495, 335–585, 357–485, 366–485, 370–495, 370–585, 503–651, 503–710) for expression and crystallization trials. The construct consisting of residues 370–495 yielded diffracting crystals.

### Cloning, expression, purification, crystallization

Clones were generated using the Polymerase Incomplete Primer Extension (PIPE) cloning method [22]. The gene encoding RF2003A.NFRKB (UniProt: Q6P4R8) was amplified by polymerase chain reaction (PCR) using a *Homo sapiens* cDNA from the Mammalian Gene Collection (MGC) as template, *PfuTurbo* DNA polymerase (Stratagene) and I-PIPE (Insert) primers (forward primer, 5′-ctgtacttccagggcCTTGGAATCAATGAAATATCTTCCAGC -3′; reverse primer, 5′-aattaagtcgcgttaTGAGCTGTCTTCATTTTCTTGCTTACAG-3′, target sequence in upper case) that included sequences for the predicted 5′ and 3′ ends of the full length construct. The expression vector, pSpeedET, which encodes an amino-terminal tobacco etch virus (TEV) protease-cleavable expression and purification tag (MGSDKIHHHHHHENLYFQ/G), was PCR amplified with V-PIPE (Vector) primers (forward primer: 5′-taacgcgacttaattaactcgtttaaacggtctccagc-3′, reverse primer: 5′-gccctggaagtacaggttttcgtgatgatgatgatgatg-3′). V-PIPE and I-PIPE PCR products were mixed to anneal the amplified DNA fragments together. *Escherichia coli* GeneHogs (Invitrogen) competent cells were transformed with the I-PIPE/V-PIPE mixture and dispensed on selective LB-agar plates. The cloning junctions were confirmed by DNA sequencing. Using the PIPE method, the gene segment encoding residues M1-C369 and D496-Q1299 were deleted. Expression was performed in a selenomethionine-containing medium at 37°C. Selenomethionine was incorporated via inhibition of methionine biosynthesis [23], which does not require a methionine auxotrophic strain. At the end of fermentation, lysozyme was added to the culture to a final concentration of 250 µg/ml, and the cells were harvested and frozen. After one freeze/thaw cycle, the cells were homogenized and sonicated in lysis buffer [40 mM Tris, 300 mM NaCl, 10 mM imidazole, 1 mM Tris(2-carboxyethyl)phosphine-HCl (TCEP), pH 8.0]. The lysate was clarified by centrifugation at 32,500× g for 30 minutes. The soluble fraction was passed over nickel-chelating resin (GE Healthcare) pre-equilibrated with lysis buffer, the resin washed with wash buffer [40 mM Tris, 300 mM NaCl, 40 mM imidazole, 10% (v/v) glycerol, 1 mM TCEP, pH 8.0], and the protein was eluted with elution buffer [20 mM Tris, 300 mM imidazole, 10% (v/v) glycerol, 150 mM NaCl, 1 mM TCEP, pH 8.0]. The eluate was buffer-exchanged with TEV buffer [20 mM Tris, 150 mM NaCl, 30 mM imidazole, 1 mM TCEP, pH 8.0] using a PD-10 column (GE Healthcare), and incubated with 1 mg of TEV protease per 15 mg of eluted protein for 2 hr at ambient temperature followed by overnight incubation at 4°C. The protease-treated eluate was passed over nickel-chelating resin (GE Healthcare) pre-equilibrated with crystallization buffer [20 mM Tris, 150 mM NaCl, 30 mM imidazole, 1 mM TCEP, pH 8.0] and the resin was washed with the same buffer. The flow-through and wash fractions were combined and concentrated to 21.3 mg/ml by centrifugal ultrafiltration (Millipore) for crystallization trials. Lysine residues were reductively methylated by adding 40 µl 0.98 M dimethylaminoborane and 80 µl 3.26% by weight formaldehyde, per milliliter of protein, over 2 hours in the presence of crystallization buffer at 277 K [24]. Methylation reagents were subsequently removed using a PD-10 column and the protein was concentrated to 26.7 mg/ml using ultrafiltration. The NFRKB_WHL construct was crystallized using the nanodroplet vapor diffusion method [25] with standard JCSG crystallization protocols [26]. Sitting drops composed of 100 nl protein solution mixed with 100 nl crystallization solution were equilibrated against a 50 µl reservoir at 277 K for 22 days prior to harvest. The crystallization solution consisted of 0.09 M HEPES pH 7.5, 10% glycerol, 1.26 M tri-sodium citrate. Ethylene glycol was added to a final concentration of 10% (v/v) as a cryoprotectant. Initial screening for diffraction was carried out using the Stanford Automated Mounting system (SAM) [27] at the Stanford Synchrotron Radiation Lightsource (SSRL, Menlo Park, CA). The complementary DNA from *Homo sapiens* (MGC Number 71524) was obtained from Invitrogen (Mammalian Gene Collection).

### Data collection, structure solution, refinement

Multi-wavelength anomalous diffraction (MAD) data were collected to 2.18 Å resolution at wavelengths corresponding to inflection (0.97936 Å), peak (0.97915 Å), and high energy remote (0.91837 Å) of the Selenium edge at beam line BL9-2 at SSRL. The data sets were collected at −173°C using a MAR325 CCD detector and the BLU-ICE data collection environment [28]. The data were processed with XDS [29] and scaled with XSCALE [30] in space group P4_3_2_1_2. Phasing was performed with SHELXD [31] and autoSHARP [32] which resulted in a mean figure of merit of 0.18 with one selenium site per protein chain. Automatic model building was performed with RESOLVE [33]. Model completion and refinement were performed with COOT [34] and REFMAC 5.6.0116 [35] using the high energy remote wavelength data. The refinement included experimental phase restraints in the form of Hendrickson-Lattman coefficients from SHARP, NCS restraints, and TLS refinement with two TLS groups per chain. Data collection and refinement statistics are summarized in Table 2.

**Table 2:** Crystallographic data and refinement statistics for NFRKB (PDB code 3u21).

	λ_1_ MAD-Se (Remote)	λ_2_ MAD-Se (Inflection)	λ_3_ MAD-Se (Peak)
**Data collection**			
Space group	P 4_3_ 2_1_ 2		
Unit cell parameters (Å)	a = b = 60.94, c = 130.84		
Wavelength (Å)	0.91837	0.97936	0.97915
Resolution range (Å)	35.5−2.18(2.26−2.18)	35.5−2.12(2.20−2.12)	36.0−2.23(2.31−2.23)
No. observations	94,617	102,509	88,204
No. unique reflections	13,560	14,695	12,703
Completeness (%)	99.8 (99.9)	99.8 (100)	99.8 (99.4)
Mean *I/σ(I)*	20.8 (2.4)	18.3 (1. 8)	21.8 (2. 2)
*R_merge_* †(%)	6.7 (79.6)	7.4 (106.6)	7.0 (87.2)
*R_meas_* ‡ (%)	7.3 (85.9)	8.0 (114.9)	7.6 (94.3)
Wilson B (Å^2^)	41.3	39.9	42.2
**Model and refinement statistics**			
Data set used in refinement (|F|>0)	λ_1_ (Remote)		
No. reflections (total)	12,842§		
No. reflections (test)	662		
Completeness (%)	99.8 (99.9)		
*R_cryst_* ¶	0.231 (0.250)		
*R_free_* ¶	0.269 (0.290)		
Ramachandran Stats (%)	97.5 (favored),0 (outliers)		
Restraints (r.m.s.d. observed)			
Bond angles (°)	1.25		
Bond lengths (Å)	0.012		
Average isotropic *B* value†† (Å^2^)	46.2		
Average B-value solvent (Å^2^)	42.7		
ESU‡‡ based on *R_free_* (Å)	0.22		
Protein residues/atoms	217/1,737		
Waters/solvent molecules	26		

Values in parentheses are for the highest resolution shell.

† R_merge_ = Σ_hkl_Σ_i_|I_i_(hkl)−(I(hkl))|/Σ_hkl_ Σ_i_(hkl).

‡ R_meas_ = Σ_hkl_[N/(N−1)]^1/2^Σ_i_|I_i_(hkl)−(I(hkl))|/Σ_hkl_Σ_i_I_i_(hkl) [41].

§ Typically, the number of unique reflections used in refinement is slightly less than the total number that were integrated and scaled. Reflections are excluded owing to systematic absences, negative intensities and rounding errors in the resolution limits and unit-cell parameters.

¶ *R_cryst_* = Σ*_hkl_*∥*F*_obs_|−|*F*_calc_∥/Σ*_hkl_*|*F*_obs_|, where *F*_calc_ and *F*_obs_ are the calculated and observed structure-factor amplitudes, respectively. *R_free_* is the same as *R_cryst_* but for 4.9% of the total reflections chosen at random and omitted from refinement.

†† This value represents the total *B* that includes TLS and residual *B* components.

‡‡ Estimated overall coordinate error [42], [43].

### DNA Binding

The Differential Scanning Fluorimetry (DSF) experiment used to measure the effect of DNA binding on protein stability was performed at room temperature using a MxPro3005P PCR instrument (Stratagene). The optimized reaction mixture contained 10 µM of NFRKB (aa 370–495), 20 µM, 80 µM or 160 µM DNA (GGGGAATCTCC; the consensus sequence for human NFRKB, Uniprot Id Q6P4R8) and 1× SYPRO Orange protein gel stain (Invitrogen) in the assay buffer (20 mM Tris-HCl pH 8.0, 10% glycerol, 5 mM DTT, 150 mM NaCl). The DNA oligonucleotides were purchased from Integrated DNA technologies. Each experiment was performed in triplicate in 96 well polypropylene plates (Agilent Technologies) by adding 20 µl of protein/DNA mixture to 30 µl of dye/buffer mixture. The reactions were mixed, centrifuged and incubated for 30 min at 4°C. For thermal stability measurements of the protein, the fluorescence of the dye was followed as a function of time using a FRROX filter set with an excitation wavelength of 492 nm and an emission wavelength of 610 nm. Data were collected from 25° to 95°C at 1°C/30 s intervals, and plotted to calculate the melting temperature of the protein (Figure 5, Table 1).

### Validation and deposition

The quality of the crystal structure was analyzed using the JCSG Quality Control server (http://smb.slac.stanford.edu/jcsg/QC/). This server verifies the stereochemical quality of the model using AutoDepInputTool [36], MolProbity [12], WHATIF [37], RESOLVE [33], as well as several in-house scripts, and summarizes the outputs. Protein quaternary structure analysis was carried out using the PISA server [38]. Figures were prepared with PyMOL [39]. Atomic coordinates and experimental structure factors have been deposited in the PDB and are accessible under the code 3u21.
